# Drought or/and Heat-Stress Effects on Seed Filling in Food Crops: Impacts on Functional Biochemistry, Seed Yields, and Nutritional Quality

**DOI:** 10.3389/fpls.2018.01705

**Published:** 2018-11-27

**Authors:** Akanksha Sehgal, Kumari Sita, Kadambot H. M. Siddique, Rakesh Kumar, Sailaja Bhogireddy, Rajeev K. Varshney, Bindumadhava HanumanthaRao, Ramakrishnan M. Nair, P. V. Vara Prasad, Harsh Nayyar

**Affiliations:** ^1^Department of Botany, Panjab University, Chandigarh, India; ^2^The UWA Institute of Agriculture, University of Western Australia, Perth, WA, Australia; ^3^Center of Excellence in Genomics and Systems Biology, International Crops Research Institute for the Semi-Arid Tropics, Hyderabad, India; ^4^World Vegetable Center, South Asia, Hyderabad, India; ^5^Sustainable Intensification Innovation Lab, Kansas State University, Manhattan, KS, United States

**Keywords:** drought stress, heat stress, photosynthates, seed filling, transcriptional regulation, omics

## Abstract

Drought (water deficits) and heat (high temperatures) stress are the prime abiotic constraints, under the current and climate change scenario in future. Any further increase in the occurrence, and extremity of these stresses, either individually or in combination, would severely reduce the crop productivity and food security, globally. Although, they obstruct productivity at all crop growth stages, the extent of damage at reproductive phase of crop growth, mainly the seed filling phase, is critical and causes considerable yield losses. Drought and heat stress substantially affect the seed yields by reducing seed size and number, eventually affecting the commercial trait ‘100 seed weight’ and seed quality. Seed filling is influenced by various metabolic processes occurring in the leaves, especially production and translocation of photoassimilates, importing precursors for biosynthesis of seed reserves, minerals and other functional constituents. These processes are highly sensitive to drought and heat, due to involvement of array of diverse enzymes and transporters, located in the leaves and seeds. We highlight here the findings in various food crops showing how their seed composition is drastically impacted at various cellular levels due to drought and heat stresses, applied separately, or in combination. The combined stresses are extremely detrimental for seed yield and its quality, and thus need more attention. Understanding the precise target sites regulating seed filling events in leaves and seeds, and how they are affected by abiotic stresses, is imperative to enhance the seed quality. It is vital to know the physiological, biochemical and genetic mechanisms, which govern the various seed filling events under stress environments, to devise strategies to improve stress tolerance. Converging modern advances in physiology, biochemistry and biotechnology, especially the “omics” technologies might provide a strong impetus to research on this aspect. Such application, along with effective agronomic management system would pave the way in developing crop genotypes/varieties with improved productivity under drought and/or heat stresses.

## Introduction

Globally, abiotic stresses drastically affect crop productivity leading to substantial yield losses. According to the IPCC (2014) report, the decline in food productivity and quality, primarily due to extreme temperatures and water deficit conditions, poses a serious threat to agriculture (IPCC, 2014; Zandalinas et al., 2018). Therefore, under the changing climate, minimizing agricultural losses caused by these stresses have become a major challenge and has created a global concern to assure food security (Anjum et al., 2011a). The effects of climate change are exacerbated by the continuous decline in availability and productivity of agricultural land (Zandalinas et al., 2018). The world is facing a gradual rise in heat wave frequency leading to warm days and nights, which is projected to exceed 2°C by the end of the 21st century (IPCC, 2014). Abiotic stresses markedly affect the reproductive development of various crops and, ultimately reduce the final economic yields. The effects of drought and high temperature stress on grain yield are complex and include processes such as nutrient assimilation and their mobilization to various reproductive organs, accumulation of stem reserves, gametogenesis, fertilization, embryogenesis, and endosperm as well as seed development. While the presence of these stresses at any growth stage can affect crop yield, the seed filling stage is crucial for determining average seed weight, seed composition and, therefore, the final quantitative and qualitative yield (Çakir, 2004; Otegui and Slafer, 2004; Prasad et al., 2017).

Stresses disrupt germination, vegetative growth, tiller production, dry matter partitioning, reproductive organ development, reproductive processes (Boyer and Westgate, 2004; Prasad et al., 2011), grain filling (Sehgal et al., 2017), and grain quality (Gooding et al., 2003; Britz et al., 2007). Reproductive processes and grain filling are more sensitive to both these stresses, and have optimum and ceiling temperatures that are relatively lower than those for vegetative growth and development phases. For instance, exposure of wheat to short episodes (2–5 days) of heat stress (>24°C) at reproductive stage (at start of heading) resulted in substantial damage to florets’ fertility, while mean daily temperature of 35° caused total failures. Rising the duration of high temperature at this stage decreased the grain weight linearly (Prasad and Djanaguiraman, 2014). Similarly, pea (Mahoney, 1991), lentil (Barghi et al., 2012) and chickpea (Wang et al., 2006) performed best at temperatures of 15–25°C at their reproductive stage, and higher temperatures (>32°C) have been found to cause pollen sterility, as in chickpea (Kaushal et al., 2013) and lentils (Bhandari et al., 2016). More sensitivity of reproductive stage to heat stress, compared to vegetative stage, is mainly attributed to damage to male components, which are severely impacted as a result of disruption of developmental as well as functional aspects, such as sucrose and starch accumulation in pollen grains (Sita et al., 2017). The leaves show more resilience at reproductive ceiling temperatures, probably due to some effective thermotolerance mechanisms, the differential sensitivity of two organ types, which need to be probed further.

Though both the stresses often exist in combination (Barnabás et al., 2008), yet their interactive effects on crop yield and productivity have received a little attention (Barnabás et al., 2008), apart from a few studies (Canci and Toker, 2009; Prasad et al., 2011; Hamidou et al., 2013; Awasthi et al., 2014; Sehgal et al., 2017). Not much work has been done exclusively on the impacts of dual stress on seed filling and nutritional composition (Awasthi et al., 2014; Sehgal et al., 2017). When combined, these stresses affect various molecular, biochemical and physiological functions, to more severely impair growth, quality, and yield, compared with their individual effects (Rizhsky et al., 2004; Mittler, 2006; Prasad et al., 2008a; Pradhan et al., 2012). Also, the combined heat and drought stress have distinct effects on cellular processes in plants, relative to their individual effects (Rizhsky et al., 2002, 2004; Cairns et al., 2013; Hamidou et al., 2013; Awasthi et al., 2014; Sehgal et al., 2017), suggesting stress-specific responses.

**FIGURE 1:**
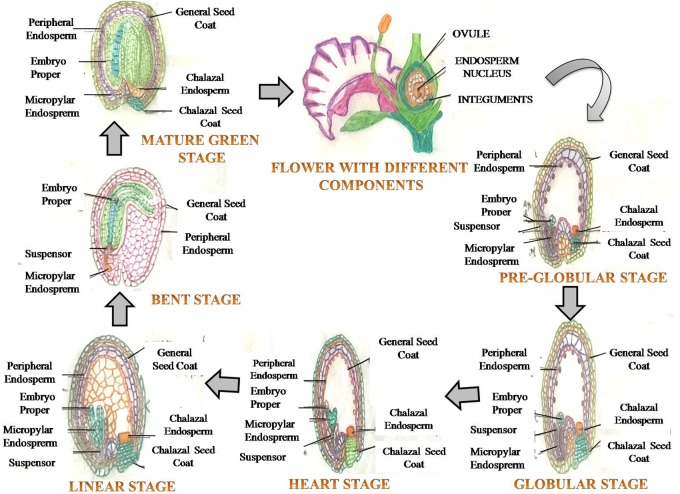
A generalized view of seed development depicting various growth stages of embryo (embryogenesis), commencing post fertilization, leading to formation of a mature seed.

**FIGURE 2:**
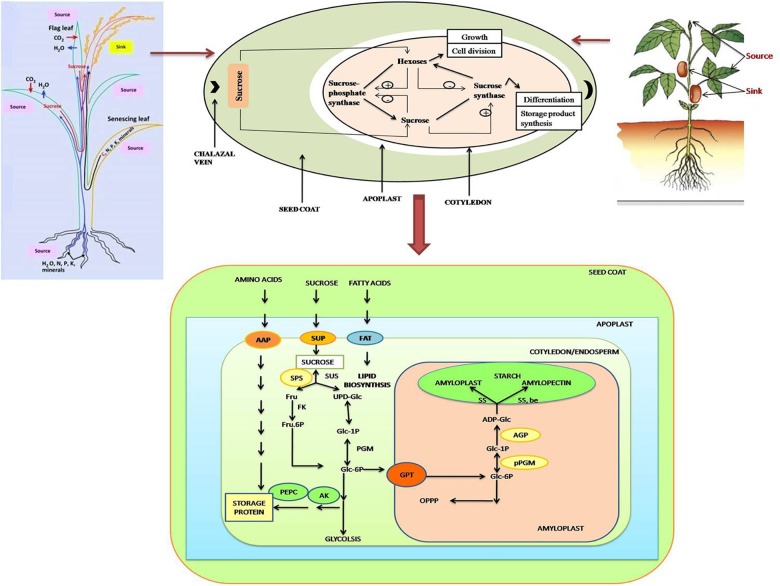
Schematic representation of various processes during seed filling stage in both monocot (top left) as well as dicot (top right) plants. The import and metabolism of sucrose is depicted in the figure. Sucrose enters the seed coat via the chalazal vein. During the pre-storage phase, sucrose is degraded through the catalytic action of cell-wall bound invertase, when the invertase activity is high leading to high ratio of hexoses to sucrose promoting growth via cell division. During storage phase of development, the invertase activity is low, so sucrose is taken up directly by the cotyledons. A low ratio of hexoses: sucrose promotes differentiation and storage product synthesis. Sucrose metabolism in the cotyledons is catalyzed by a cycle of synthesis and breakdown via sucrose-phosphate synthase and sucrose synthase. The figure also explains the translocation of sucrose and other nutrients from source (endosperm/cotyledon) to sink (embryo) during developmental stages of seed. Carbon, nitrogen, phosphorus, and other minerals, produced from the hydrolysis of stored nutrients in endosperm/cotyledon are transported to the embryo for its growth, and mobilized toward assimilate-transport pathway into developing seeds, and toward the starch and sucrose synthesis pathway. C, Carbon; N, nitrogen; P, phosphorus; K, potassium; SUT, sucrose transporter; AAP, amino acids protein; PEPC, AK, PEP carboxylase and/or aspartate kinase; AGP, ADP glucose pyro-phosphorylase; GPT, plastidic glucose-6-P translocator; pPGM, plastidic phospho-glucomutase; FAT, fatty acid transporter. Stresses such as drought and heat may affect the seed filling by influencing any of these or multiple processes to accelerate (as in heat) or disrupt (as in heat, drought) the seed filling.

**FIGURE 3:**
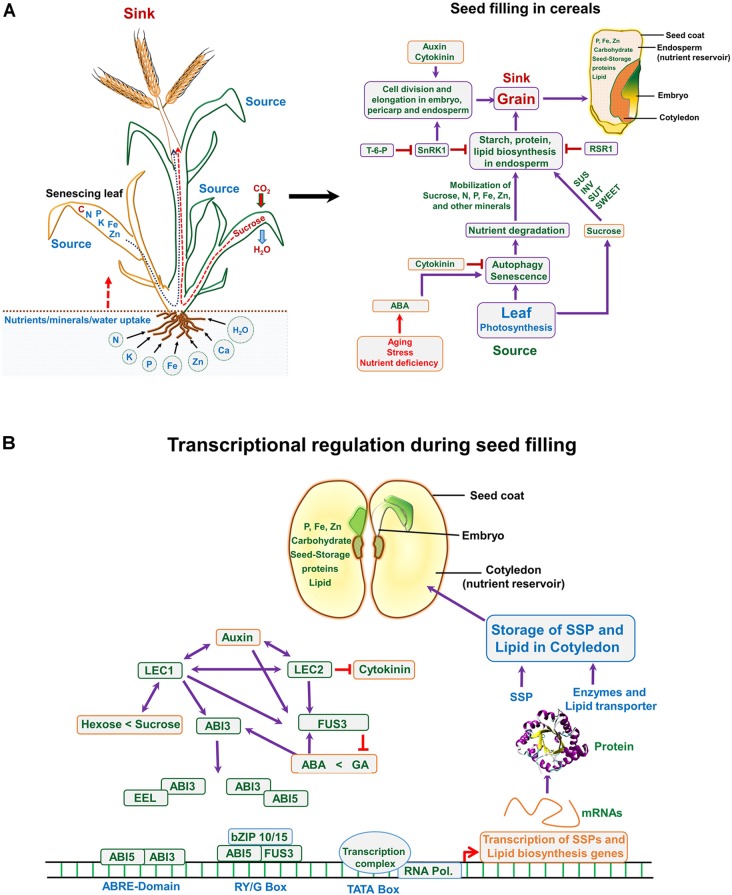
**(A)** Nutrient translocation from source to sink and a road map highlighting events associated with seed filling in monocot (cereals). **(A)** Plant takes up essential nutrients from the soil including N (nitrogen), P (phosphorus), K (potassium), Fe (iron), Zn (zinc), etc., and assimilates carbohydrate (sucrose) through fixing atmospheric CO_2_ via photosynthesis. During seed filling stage the matured leaves translocate assimilates to the developing seed (sink), whereas, nutrients especially N and other minerals are remobilized from the senescing leaves to the sink organ (developing grain). The role of hormones and cross talk between source and sink during seed filling; at seed filling stage, stress hormones serve as key factors, which control the autophagy and senescence, thus translocating the N-pool and the minerals from the senescing leaves to the grain/seed. Auxins and cytokinins are important and regulate the seed cell numbers and size, this controlling the sink strength. **(B)** In dicots, the seed development and filling is controlled through transcriptional regulation. Several transcription factors interact/overlap with each other and also involve hormonal control during this event, as indicated in the figure. ABA, abscisic acid; GA, gibberellic acid; T-6-P, trehalose-6-phosphate; LEC1, leafy cotyledon 1; ABI3, abscisic acid-insensitive 3; EEL, enhanced em level; fusca3, FUS3; SnRK1, SNF1-related protein kinase; SUS, sucrose synthase; SUTs, sucrose transporters; IVT, invertase; RSR1, rice starch regulator 1; SSP, seed storage protein.

**FIGURE 4:**
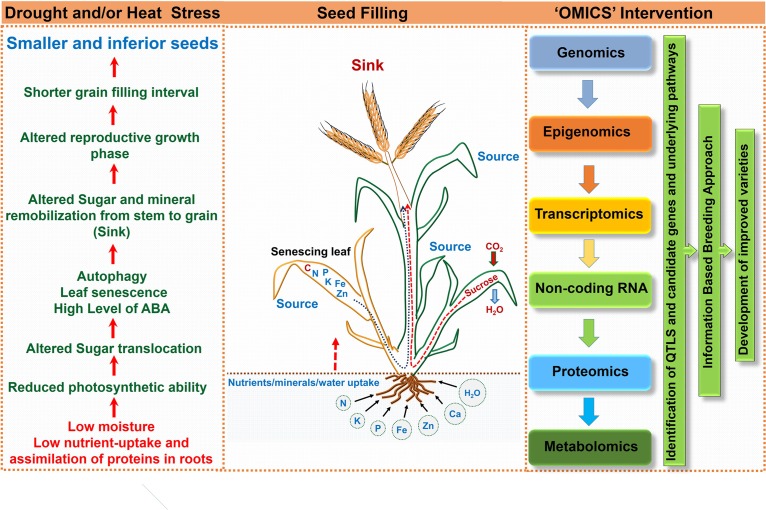
Schematic representation highlighting consequences of drought and/or heat stress on seed filling and ‘omics’ approach for crop improvement.

Seed filling is a crucial growth stage for all crops, which involves mobilization and transport processes required for importing various constituents, and many biochemical processes for the synthesis of proteins, carbohydrates and lipids in the developing seeds (Barnabás et al., 2008; Prasad et al., 2008a; Awasthi et al., 2014; Farooq et al., 2017a) (Figures 1, 2). Seed filling processes and the accumulation of reserves in the developing and maturing seeds are highly sensitive to environmental changes, which influence the qualitative and quantitative traits of the final yield (Yang and Zhang, 2006) (Figures 3, 4). Heat and drought stress can hinder the accumulation of various seed constituents, primarily starch and proteins (Behboudian et al., 2001; Asthir et al., 2012; Farooq et al., 2017a,b) (Figure 3) through inhibiting the enzymatic processes of synthesis of starch (Ahmadi and Baker, 2001) and proteins (Triboï et al., 2003) (Figure 2).

Here, we initially describe the process of seed filling, and later, illustrate how drought and heat stress, either separately and in combination, impact seed yield and quality, and also attempt to explain the advancement in the ‘omics’ technology in addressing these vital challenges. We believe, our efforts would enable the researchers/breeders to evolve strategies to develop stress-resilient, high yielding, and nutritionally superior crops for the future.

## Seed Development and Filling/Maturation

In angiosperms, seed filling is the terminal stage of growth in plants where fertilized ovaries form caryopses/inflorescences, and involves several processes related to the import of constituents, and biochemical processes associated with carbohydrate, protein and lipids synthesis in seeds (Dante et al., 2014). The rate and duration of seed filling affect the final seed weight (seed size), a primary component of total seed yield. The seed originates from a double fertilization event, leading to the formation of a triploid endosperm and diploid zygote (Yang and Zhang, 2006). The seed develops from the ovule, and has embryo and endosperm/cotyledon, surrounded by the maternally derived seed coat that develops from integuments of the ovule (Figure 1). The primary function of the seed is to protect the embryo, sensing favorable conditions for germination and nourishment of the germinating seedling (Emes et al., 2003). The embryo embodies the structures of the future adult plant (Locascio et al., 2014), and encloses the essential elements and basic processes for the development of a new plant (Locascio et al., 2014; Paula et al., 2016). Seed expansion involves rapid and early cell division of the zygote and triploid nucellus. The division of cells is accompanied by intake of water, which further leads to cell extension (Altenbach et al., 2003; Emes et al., 2003) (Figure 1). Cell expansion and division starts with more uptake of water, and when cell expansion is completed, cells are destined to maturity (Figure 1). After 2–3 weeks of anthesis, cell division in cotyledons/endosperm halts with net rise in moisture content per seed/grain, which represents maximum seed size (Schnyder and Baum, 1992). Moisture content is a key factor controlling seed development, seed filling and metabolic activity of developing seed. The processes related to synthesis and accumulation of various seed reserves are largely influenced by the moisture status of the storage cells, depletion of water at this stage disrupts the seed filling (Ochatt, 2015). The duration of seed filling is inversely proportional to moisture loss and biomass deposition (Gambín et al., 2007). Seed development has been excellently described recently, particularly in legumes (see Ochatt, 2015; for more details).

## Photosynthates Assimilation During Seed Filling

The quantitative and qualitative characteristics of yield are strongly affected by seed filling process and nutrient reserve accumulation in both developing as well as maturing seeds, and both are responsive to environmental conditions (Yang and Zhang, 2006; Barnabás et al., 2008) (Figures 2, 3). Seed filling in plants depends upon two sources, transfer of current assimilates directly to seeds and its redistribution from vegetative reserve pools, either pre- or post-anthesis stage (Yang and Zhang, 2006) (Figures 2, 3). These reserve pools provide substrates essential to maintain transport and supply of assimilates to seeds all through the dark phase of the diurnal cycle, as well as, for duration of the later seed-filling period, when photosynthetic apparatus is becoming senescent, and dry matter accumulation rate of grains exceed dry matter accumulation rate of whole plant (Schnyder, 1993). Under normal condition, assimilate reserves during pre-anthesis in stems and sheaths of rice (*Oryza sativa*) and wheat (*Triticum aestivum*) contribute around 10–40% to the final seed weight (Gebbing and Schnyder, 1999). Remobilization of these reserves to the seed becomes vital in determining seed size on exposure of plants to unfavorable environment or if yield potential is dependent on high biomass accumulation (Asseng and van Herwaarden, 2003; Plaut et al., 2004; Yang and Zhang, 2006). The contribution of assimilates supply from stem reserves may increase up to 40% during heat and drought stress situations (Bidinger et al., 1977; Gebbing and Schnyder, 1999). Drought reduces photosynthesis, hence source strength; moreover the turgor in phloem cells is also reduced by water deficiency, thereby increasing the viscosity of sucrose to inhibit its flow through the conducting cells toward the sinks (seeds) (Sevanto, 2014). Combined stresses may result in more dehydration to severely slow down the phloem transport (McDowell et al., 2013). Sucrose transporters (SUTs) have a vital role in the export of sucrose from the leaves to the sinks, the expression of *SUT* genes is altered by drought (mild–severe) in soybean, barley, wheat, and maize (Xu et al., 2018); down-regulation of these transporters has been reported in some cases (Xue et al., 2016). On the other hand, the pattern of *SUT* expression in heat-stressed plants differs from the drought-stressed plants. Arabidopsis *AtSUT2* is down-regulated under heat stress (about 15°C increase), while it is up-regulated under drought (Xu et al., 2018). In contrast, *PtaSUT4* (a symplastic loader poplar) is up-regulated under heat stress (17°C increase), while down-regulated under drought stress, which correlates with reduced sucrose transport from the leaves (Xue et al., 2016). Drought resulted in fivefold reduction in cytosolic acid invertase activity in Lupin seeds, suggesting that the amount of sucrose available is reduced in seeds (Kim et al., 2000). Heat stress down-regulated *OsSUT1* in rice stems, more so in a sensitive cultivar, to reduce the grain quality (Miyazaki et al., 2013; Phan et al., 2013). In barley too, several genes involved in sucrose and starch biosynthesis were repressed by heat stress, along with down-regulation of SUTs (*HvSUT1* and *HvSTP3*) (Mangelsen et al., 2011). Thus, photo-assimilation is disrupted because of down-regulation of SUTs in stressed plants. Similar studies on plants subjected to combined drought and heat need to be conducted to find out the types of transporters affected.

## Hormonal Control During Photosynthate Partitioning and Seed Filling

Seed expansion and filling include all processes involved in formation and structural development of mature seed (Locascio et al., 2014). Seed-making process is highly coordinated and complex, (Figures 1, 2) requiring hormonal regulation (Ochatt, 2011) (Figure 3), and a constant exchange of signals from-and-to maternal tissues, and between cotyledon/endosperm and embryo (Locascio et al., 2014). The continual interaction among these three constituents of seed ensures synchronized seed development (Locascio et al., 2014). Many reports showed substantial changes in hormones content during seed development and filling. Auxin is linked with maize grain filling through controlling cell wall-associated invertase enzyme activity. The increase in auxin during seed filling interval accompanied with increased grain filling rate. The increased auxin is known to enhance sink capacity through enlargement of cell and increased nutrient assimilation (Kong et al., 2015). Abscisic acid (ABA), auxin, and cytokinins (CKs) play major role in grain filling through source photosynthate/nutrient re-mobilization and grain development in cereals (Yu et al., 2015). Notably, the source activity and sink strength are synchronized and can be altered by hormones and external/environmental stimulus. The grain filling is closely associated with senescence and senescence-related hormone ABA, affecting the time for nutrient mobilization and grain filling in barley, wheat, rice, and sorghum (Yu et al., 2015). In barley, ABA can induce the orchestration of gene expression of senescence-related genes (Yang et al., 2003). Such activities are also in action during drought stress to accelerate seed filling. In rice and wheat, water stress at grain filling stage caused reduced photosynthetic activity and induced accelerated leaf senescence, resulting in shortened grain filling period due to enhanced ABA level and remobilization of carbon (C)-pool from stem to leaves (Yang et al., 2003; Yang and Zhang, 2006). Drought-tolerant plants exhibit delayed leaf senescence, thus cultivars with stay-green trait having advantage over terminal drought (Jordan et al., 2012), which is beneficial to sustain seed filling. Early onset of leaf senescence causes inadequate nutrient supply to grains during grain filling, whereas, late initiation of nutrient mobilization may not support rapid developing seed. This synchronized developmental program is known to involve ABA-inducible NAC-transcription factor (TF), which regulates the expression of genes involved in mineral-nutrient remobilization from leaf to grain, chlorophyll degradation and leaf senescence in rice (Uauy et al., 2006). A reduced expression of this ABA-regulated NAC – TF caused enhancement in yield in the transgenic rice, plausibly due to prolonged supply of photosynthates/nutrients into the developing grains through fine-tuned ABA biosynthesis pathway. CK also play a key role in grain filling by initiating rapid cell division of endosperm cells (Kong et al., 2015). The increased level of CKs probably enhances sink strength by up-regulating cell division related genes via sugar signaling, which involves enhanced phloem unloading and sugar import to endospermic cells through a cell wall-associated enzyme invertase (Rijavec et al., 2009). Overproduction of CK delays early senescence through reduced proteolytic activity and inhibition of N-remobilization, which increases life span and confers drought tolerance in crop plants (Gregersen et al., 2013). Thus, seed filling is determined by diverse plant hormones having specific functions, their interplay might be vital in regulating various processes related to accumulation of seed reserves (Ochatt, 2015).

## Impact of Abiotic Stresses on Seed Filling

Drought stress during the initial stage of seed development reduces ability of kernel/seed sink strength by decreasing the number of endosperm cells and amyloplasts formed (Saini and Westgate, 2000), thus reducing grain weight with a decline in endosperm competence to gather starch, in terms of both rate and duration (Nicolas et al., 1985). Likewise, heat stress can significantly influence seed development and thus decreases seed yield in several crops including cereals (Prasad et al., 2008a; Dias and Lidon, 2009), legumes (Prasad et al., 2002; Awasthi et al., 2014; Bhandari et al., 2016; Sharma L. et al., 2016; Sehgal et al., 2017). Seed filling is closely related to the process of whole-plant senescence (Yang and Zhang, 2006). Usually, drought and heat stress during seed filling causes early senescence and reduces seed-filling duration, and enhances assimilate remobilization from the source to sink (Asseng and van Herwaarden, 2003; Plaut et al., 2004), the combined effects are more severe (Awasthi et al., 2014). The stress-induced reduction in assimilate supply strongly influences grain development (Figure 2) (Sharkey, 2005; Subramanyam et al., 2006; Farooq et al., 2009). Here, we describe how drought or/and heat stresses impact the process of seed filling and ultimately influence seed yield and its quality, citing many definite examples from various crop species, through findings at changes in physiology, biochemistry, proteins, and genes.

## Drought Affects Overall Plant Growth and Nutritional Status of Plant Affecting Seed Filling

Drought stress limits vegetative growth by reducing leaf water content in various cereals (Siddique et al., 2001; Valentovic et al., 2006) and legumes (França et al., 2000), which might be markedly influenced by inhibition of stomatal conductance/transpiration (Anjum et al., 2011b). Reduced stomatal conductance resulted in increase in leaf temperatures (Sehgal et al., 2017), both of which induced leaf wilting (Farooq et al., 2009, 2017a). Drought stress can cause membrane damage (Jiang and Huang, 2001; Awasthi et al., 2014), chlorophyll (Massacci et al., 2008; Rahbarian et al., 2011) and photosynthesis (Samarah et al., 2009a; Anjum et al., 2011b), due to stomatal or non-stomatal associated mechanisms. Drought stress impairs mineral uptake (Samarah et al., 2004; Gunes et al., 2006) and drastically reduces nitrogen fixation in legumes such as in soybean (Serraj, 2003), and pea (Gonzalez et al., 2001). Collectively, these adverse effects eventually decrease assimilate production and mobilization to developing seeds in various crops (Leport et al., 2006; Mafakheri et al., 2010; Zare et al., 2012).

## Drought Stress Limits Reproductive Phase and Seed Development Affecting Seed Yield

The reproductive stage of growth is more sensitive to drought than the vegetative stage, resulting in fewer flowers, poor pod or fruit set, which decreases seed numbers (Seghatoleslami et al., 2008; Pushpavalli et al., 2014). Gametogenesis, fertilization, embryogenesis are several impacted, to seriously limit seed development thereby lowering crop yields (Farooq et al., 2009, 2014). Flowering and reproductive developmental stages are among the most disrupted stages during drought stress (Samarah et al., 2009a; Fang et al., 2010). Pollen sterility (Al-Ghzawi et al., 2009) is a common symptom, which decreases pollen germination, hinders pollen tube growth to impair fertilization and reduce seed yield (Fang et al., 2010; Gusmao et al., 2012). Stress because of limited water causes carbohydrate deprivation, elevated endogenous ABA levels, and a reduced ability of reproductive sinks to use sucrose and starch. The increase in non-reducing sugars and failure to accumulate starch under drought stress results in ovary abortion, leading to poor grain set and grain yield (Andersen et al., 2002). A reduction in tissue water potential decreases the activity of acid invertase (a vital enzyme in seed development) in seeds, which inhibits sucrose import (Farooq et al., 2015). Thus, scarce energy sources and elevated ABA levels result in poor grain set under drought stress (Liu et al., 2004). Seed yield was drastically reduced in crops exposed to drought stress at the time of seed filling, for example in several legume crops (Shrestha et al., 2006; Ghanbari et al., 2013a; Awasthi et al., 2014). Moreover, drought stress at the early phase of seed filling decreased the subsequent germination percentage (approximately 9%) of the progeny in soybean (*Glycine max* L.), as compared to control plants (Smiciklas et al., 1992). Similarly, Dornbos and Mullen (1985) reported a 5% decline in seed germination, 12% decline in seedling vigor and 19% increase in electrical conductivity of seed leachate in soybean seeds obtained from drought-stressed plants. In peanut (*Arachis hypogaea* L.), drought stress during seed development moderately reduced seed germination, without any impact on seedling vigor (Ketring, 1991). In chickpea, the medium-sized seeds produced under drought stress have lower germination rates and reduced seed vigor as compared to the control or non-irrigated seedlings (Samarah et al., 2009b). Thus, drought stress at reproductive stage inhibits the production of seeds because of disruption of gamete development and function while at seed filling stage limits the seed size because of inhibitory effects on carbohydrates involving phytohormones ABA.

## Drought Stress Affects Enrichment of Carbohydrates During Seed Filling Impacting Seed Size and Quality

Drought stress during seed filling slows down the seed-filling rate and reduces the filling duration to limit seed size (Fang et al., 2010; Moradi et al., 2013; Sehgal et al., 2017). It inhibits cell division in endosperm cells and number of starch granules in grains to reduce their (grains) size (Nicolas et al., 1985). Water stress markedly affects grain composition (Behboudian et al., 2001). For instance, starch content of developing wheat grains declined markedly, when plants were subjected to drought stress, which was attributed to an insufficient supply of photoassimilates, or to direct effects on starch synthesis machinery in grains, influenced by sink dehydration (Ahmadi and Baker, 2001), reduction in endosperm cell numbers, small size of starch grains (Nicolas et al., 1985), and lower amylase contents in starch grains (Singh et al., 2008). Starch accumulation involves several complex enzymatic processes, with AGPase, soluble starch synthase (SSS), and starch branching enzyme (SBE) having vital roles (Morell et al., 2001). A gene expression study in wheat showed that drought stress reduced the transcript number for the SSS enzyme more than, other enzymes involved in starch biosynthesis (Hurkman et al., 2003). In another study in wheat plants, exposed to drought stress during grain filling, glucose, fructose, and sucrose levels declined significantly in grains of a drought-sensitive genotype, which was accompanied by a sharp reduction in activities of cell wall invertase and soluble invertase (Saeedipour, 2011). Higher sucrose synthase activity in drought-tolerant variety enhances supply of assimilates to grains to increase the seed size (Saeedipour, 2011). Various enzymes involved in starch and sucrose metabolism were inhibited to reduce seed starch in chickpea (Awasthi et al., 2014). The enzymatic activities of SSS, granule-bound starch synthase (GBSS), SBE and starch de-branching enzymes (DBE) decreased to different extents in sorghum grains, which inhibited starch accumulation (Bing et al., 2014). Sugar metabolism was also inhibited in grains of drought-stressed maize, as indicated by decreased soluble and insoluble invertases activities (Zinselmeier et al., 1995, 1999). In general, because of water limitation, cell number in endosperm or cotyledons decreases, which may occur due to inhibited cell division, however, the finer mechanisms controlling these events need to be probed. Carbohydrates’ accumulation is hampered because of limitations in availability of sucrose from leaves, as well as their synthesis in developing seeds, and/or because of enzymatic inhibitions, to eventually decrease the seed size.

## Drought Stress Affects Lipid Enrichment During Seed Filling

Seeds store carbon for energy, particularly in form of lipids in oil seeds, which are mainly required for germination. Drought stress also affected oil content and quality, linoleic acid and behenic fatty acid contents declined while stearic and oleic fatty acid contents increased in drought-stressed plants of peanuts (Dwivedi et al., 1996). There are contrasting reports too, which are possibly due to variations in imposed stress treatments. For instance, in maize, drought stress markedly decreased seed oil content but enhanced linolenic acid and oleic acid contents in oil (Ali et al., 2012). There was a reduction in overall seed oil, total tocopherols, flavonoids, and oil phenolics (Ali et al., 2010, 2012). Drought stress in soybean, during seed filling, decreased oil content up to 12.4%, along with a reduction in oleic acid content (Dornbos and Mullen, 1992). The oil content is affected due to decrease in concentration of digestible carbohydrates such as glucose, fructose, and sucrose under drought conditions, which affects the fatty acids composition due to reduced unloading of sugars from stem to developing seeds (Bellaloui et al., 2013). More research is needed to explore the target sites of drought stress in pathways involved in lipids biosynthesis and how lipid quality is regulated under drought stress also requires to be investigated.

## Drought Stress Affects Nitrogen Assimilation to Result in Poor Quality Seeds

Drought stress impairs symbiotic nitrogen fixation through rising oxygen diffusion resistance to root bacteroides resulting in reduced activity of nitrogenase that decreases nitrogen availability for biosynthesis of proteins, which is a primary reserve in grain legumes, and reduces seed yield (Purcell and King, 1996). While seed protein quality largely depends on the genotype, it may be influenced by environmental stresses (Triboï et al., 2003). Alterations in composition of protein fraction due to drought and heat stress are primarily due to changes in quantity of total nitrogen accumulated during seed filling (Triboï et al., 2003). Some studies in legumes have indicated reduction in accumulation of minerals in developing seeds due to drought stress. For example, in common bean, Fe, Zn, P, and N concentrations decreased under drought stress, which correlated with reduction in total protein content (Ghanbari et al., 2013b). In another study, in white, red and ‘chitti’ bean cultivars, drought-stress during pod filling resulted in decline in seed nitrogen and protein content, substantially (Ghanbari et al., 2013a). A marked reduction in starch, protein, and amino acid contents occurs in chickpea seeds, drought-stressed during seed filling, more impact occurs on ‘Kabuli’ than ‘Desi’ types (Nayyar et al., 2006). The free amino acid pool increased but protein-amino acid fraction decreases in cowpea seeds of drought-stressed plants, and inhibition in incorporation of amino acids into protein chain occurs (Labanauskas et al., 1981). Proteomic studies in wheat grains, harvested from drought-stressed plants at grain filling stage, indicated a marked change in quality of seed proteins. While globulin and glutenin, remained unaffected, albumin and gliadin concentrations increased significantly under drought conditions (Zhang et al., 2014). In contrast, some other studies reported increased concentrations of seed protein in response to drought stress in various crop species, for example, in cereals (Gooding et al., 2003), legumes (Behboudian et al., 2001; Teixeira and Pereira, 2007) and oil seeds (Bouchereau et al., 1996). These differences may be attributed to intensity and duration of drought stress imposed on plants, and relative to seed dry weight. The increased protein content is linked to altered C-partitioning, especially in cereals, which changes the C/N ratio, to favor more N-assimilation. Thus, these studies indicate crop-specific effects of drought stress on proteins and minerals. Further work is required to investigate the impacts of drought stress on various classes of proteins, amino acid composition, especially in food legumes. Additional work should focus in future on seed quality aspects, especially on mineral transport mechanisms in seeds of drought stressed crops.

## Deleterious Effects of Heat Stress on Seed Filling

Heat stress affects almost all the stages of the plant beginning from germination until maturity. Exposure to heat stress during pod and seed filling stages results in a substantial decrease in economic yield of crop plants by reduction in seed weight. Decline in seed weight and seed number due to high temperatures has been reported in several crops including legumes (Prasad et al., 2000; Tsukaguchi et al., 2003; Devasirvatham et al., 2010), cereals (Djanaguiraman et al., 2010) and others (Rashid et al., 2017). Sustaining grain weight in heat stress conditions during seed filling stage is considered a part of heat stress tolerance mechanism (Tyagi et al., 2003; Hasanuzzaman et al., 2013). The sensitivity of seed filling to heat stress may differ according to different crop species (Sung et al., 2003; Kaushal et al., 2016). Two traits mainly seed filling rate and potential seed weight may be considered as a selection criteria for heat stress tolerance (Dias and Lidon, 2009). High-temperature stress may speed up the rate of seed filling by reducing the duration of this stage and therefore the yield potential (Prasad et al., 2011; Kaushal et al., 2016). Increase in seed-filling rate resulted in smaller and wrinkled seeds in chickpea (Kaushal et al., 2013) and lentil (Sita et al., 2017), which mainly occurred due to reductions in the remobilization and translocation of photosynthesis to developing seeds (Farooq et al., 2017b). The time of seed filling reduced in pea, soybean and white lupin, resulting in smaller grains (Duthion and Pigeaire, 1991). Specifically, in cowpea, increasing the temperature from 15.5 to 26.6°C decreased seed-filling duration from 21 to 14 days (Nielsen and Hall, 1985) and in chickpea, a 1.7°C increase in temperature reduced seed-filling duration, and accelerated maturity, which decreased seed yield (Chakrabarti et al., 2013). The reduction in seed size was related to structural and functional reasons. The cotyledon cell number and cell expansion decreased under heat stress, which inhibits the rate of seed-filling and seed size (Munier-Jolain and Ney, 1998). ABA levels were altered, which correlated with adverse effects on seed filling rate in heat-stressed chickpea (Munier-Jolain and Ney, 1998). Studies in future need to focus on probing the role of various phytohormones in altering the rate of seed filling as well as structural changes in endosperm and cotyledons in seeds developing under heat stress environment, involving tolerant and sensitive genotypes of various crops.

## Association of Seed Filling Duration With Leaf Senescence and Source–Sink Flux During Heat Stress

High temperatures during seed filling may stimulate leaf senescence to reduce photosynthetic capacity, which impacts seed development and reduces growth and yield traits in grain legumes (Farooq et al., 2017b; Sita et al., 2017). Senescence occurs due to disruption of cellular structure and organization, with a substantial reduction in chlorophyll, which also terminates the photosynthetic process (Ougham et al., 2008). Besides, causing senescence, increased temperature accelerates scorching and abscission of leaves (Ismail and Hall, 1999). Heat stress decreases photosynthetic activity and induces premature senescence, which decreases synthesis and distribution of assimilates to seeds (McDonald and Paulsen, 1997). A rise in temperature (30–35°C) for a few days repressed photosynthesis and electron flow, interrupted metabolic pathways, damaged seed set and seed development, and ultimately reduced seed yield in chickpea (Gaur et al., 2015). Rise in temperature inhibited sucrose metabolism in leaves and impaired sucrose supply to developing seeds, as in chickpea (Kaushal et al., 2013), mung bean (Kaur et al., 2015), which might be a primary reason of shriveled seeds under heat stress. Moreover, the rate of chlorophyll decline from the leaf was strongly coordinated with contents of non-structural carbohydrates and nitrogen as well as their remobilization efficiencies (Tahir and Nakata, 2005; Prasad et al., 2008a). Damage to membranes of leaves is a common effect of heat stress, along with increase in ethylene production (Djanaguiraman and Prasad, 2010), which may accelerate the senescence. Heat stress increases leaf senescence by disrupting chloroplasts and damaging chlorophyll due to direct or indirect mechanisms such as photo-oxidation, which severely inhibits photosynthetic ability to reduce biomass and seed yield (Farooq et al., 2017b). Moreover, carbon fixation is inhibited severely by heat stress due to reduction in activities of PEP carboxylase and RuBP carboxylase in photosynthetic organs during seed filling, as observed in wheat (Xu et al., 2004). These studies collectively indicate that heat-stresses induces leaf senescence, inhibits net photosynthetic rate, chlorophylls, hastens seed filling, disrupts sucrose-starch conversion and causes loss of sink activity to decrease the seed weight and quality.

The seed filling rate in plants is mainly dependent on two carbon resources (1) currently synthesized assimilates from photosynthesis, and (2) carbohydrate (assimilates) reserves translocated to the seed from vegetative tissue in leaves and stem (Plaut et al., 2004; Yang and Zhang, 2006). Impairment of photosynthesis during heat stress (Subramanyam et al., 2006), reduces the currently available assimilates to the seed. Thus, stem reserves’ mobilization play a crucial role (Blum et al., 1994); hence, the stored carbohydrates become the chief source of transported materials, contributing around 75–100% to grain yield during stress environment (Farooq et al., 2017b). The mobilization of stored reserves from leaves and stem strongly correlates with carbohydrate metabolism, involving synthesis of sucrose and its utilization (Prasad et al., 2008b). More studies are needed to explore the genotypes of various crops having ‘Stay-green’ ability in leaves under heat stress to sustain seed filling (Abdelrahman et al., 2017).

## Altered Levels of Starch, Sucrose, and Other Sugars Levels During Seed Filling Phase in Response to Heat Stress

Over 65% of seed dry weight is accounted for by starch (Barnabás et al., 2008), therefore, decrease in seed yield is mainly caused by decline in starch accumulation. Heat stress during grain filling markedly decreased starch accumulation in wheat (Hurkman et al., 2003) and rice (Yamakawa and Hakata, 2010) by altering the expression of starch-related genes, which contributed toward reduction in seed size (DuPont and Altenbach, 2003). Total non-structural carbohydrates also decreased, which changed the proportion of soluble sugars to starch (Thomas et al., 2003). Sugars such as fructose, sugar nucleotides and hexose phosphate levels also declined due to heat stress, as in wheat (Jenner, 1991). The decrease in sugars in heat-stressed plants may be related to enhanced assimilate utilization rather than production (Asthir et al., 2012). In some cases, increase in sugar levels was reported, which was related to up-regulation of starch hydrolyzing enzymes such as α-amylase during seed filling (Yamakawa et al., 2007; Tanamachi et al., 2016). Moreover, due to enhanced activity of the α-amylase enzyme, rice produce chaffy grains under heat stress environment. The transcripts of the β-glucosidase gene decrease due to heat stress to impact the seed composition in soybean (Thomas et al., 2003). Thus, heat stress adversely affects the accumulation of carbohydrates by influencing their metabolic pathways, the changes are crop-specific, and depend upon exposure to heat stress. Comprehensive studies are needed in the future involving SUTs and various genes related to starch-sugar inter-conversions in seeds of heat-stressed plants of various crops to know the accumulation patterns of various carbohydrates.

## Modulation of the Endosperm Morphology During Seed Filling Phase Under Heat Stress Periods

The amount of starch and proteins, accumulating in each seed, depends on total number of endosperm cells, determined early in stage of seed filling, and the final size of cells, which is regulated by the rate and duration of seed filling (Egli, 1998; Farooq et al., 2017b). Heat stress during early seed development reduced the endosperm cell number (Nicolas et al., 1985), however, at the later stage, heat stress impaired starch synthesis either due to limited supply of assimilates to seeds (Blum, 1998) or as a result of the direct effects on biosynthesis processes in the seed (Yang et al., 2004; Barnabás et al., 2008). Endosperm structures were altered and storage products were degraded due to heat stress (35°C for 5 days) during the seed filling in barley (Wallwork et al., 1998) and maize seeds while in rice seeds, heat stress accelerated the growth rate of endosperm resulting in chalky grains (Mitsui et al., 2016). In dicots like soybean, heat stress negatively affected cotyledon cell number, cell expansion, and hence seed filling rate, which reduced seed weight (Munier-Jolain and Ney, 1998). Thus, heat stress limited the production of storage cells in monocots and dicots, which further restricted the accumulation of various seed reserves. The mechanisms affecting the cell number in endosperm and cotyledons in seeds of heat-stressed plants are not well understood, an elaborative study is in need.

## Alterations of Storage Proteins and Lipids During Seed Filling Phase Under Heat Stress Episodes

An enhanced rate of seed filling under heat stress may be due to increased enzyme and metabolic processes, which accelerates and compresses the overall process of seed development at elevated temperatures. Increase in rate of seed dry matter accumulation may be a compensation for the decline in its duration (DuPont and Altenbach, 2003). Heat stress decreased the duration and amount of protein accumulation, but the rate of accumulation was unaffected, as in some studies (Stone et al., 1997). The storage protein composition was also altered under heat stress due to changes in the amount of total nitrogen accumulated during seed filling in wheat (Triboï et al., 2003; Barnabás et al., 2008). Protein quality deteriorated in heat-stressed plants, for example, dough quality declined in wheat under heat stress, because of reduction in aggregation properties, caused by a decline in high molecular weight glutenins, and rise in gliadin accumulation (Stone et al., 1997). Moreover, the ratio of gliadins and glutenins increased while the ratio of large polymers decreases in wheat grain flour (Panozzo and Eagles, 2000; DuPont and Altenbach, 2003). Similarly, in pea, high temperature denatured and aggregated the seed storage proteins (globulins, legumin, and vicilin) in pea (Mession et al., 2013); which might be due to loss of covalent and non-covalent interactions (Sun and Arntfield, 2012). In the same way, heat stress denatured β-conglycinin in soybean (Iwabuchi and Yamauchi, 1984) and damaged globulin and phaseolin (Hernández-Unzón and Ortega-Delgado, 1988). Seed protein fractions, especially albumins and globulins were adversely impacted by heat stress in lentil seeds (Sita et al., 2018), thus reducing the overall seed quality. The damage to the structure and metabolism of proteins due to heat stress also inhibited the activities of enzymes involved in protein synthesis, as in Andean lupin (*Lupinus mutabilis*) (Zu, 2009).

Fatty acid composition and content are variably affected by heat stress in a crop-specific response. For instance, different temperatures (10, 16, 21, 26.5°C), imposed on plants of rapeseed (*Brassica napus*), safflower (*Carthamus tinctorius*), flax (*Linum usitatissimum*), sunflower (*Helianthus annuus*) and castor bean (*Ricinus communis*) plants, during seed development, did not affect the composition of fatty acid in the oil of safflower and castor bean, but reduced the amounts of unsaturated fatty acids (Canvin, 1965; Brunel-Muguet et al., 2015). The oil composition changed because of the impact of heat stress on fatty acid biosynthesis in sunflower (Harris et al., 1978) and in soybean, heat stress (35°C) during seed-filling decreased oil content by 2.6%, compared with seeds from plants exposed to 29°C (Dornbos and Mullen, 1992). Considering that, heat stress effects are likely to be more prominent in future, it would be vital to understand the target sites of heat stress in the biochemical pathways, related to accumulation of proteins and lipids in developing seeds of various food crops.

## Combined Effect of Heat and Drought Stresses on Seed Filling

Both drought and heat stress, when applied jointly, reduce leaf water content more severely, resulting in early leaf wilting, acute chlorosis and membrane damage (Awasthi et al., 2014), drastic inhibition of photosynthesis and production of assimilates in leaves was recorded by various studies, for instance, in chickpea (Awasthi et al., 2014); lentil (Sehgal et al., 2017; Sita et al., 2017) and barley (Roohi et al., 2013). Though the impact of each of this stress, when applied singly on plants may differ, depending upon its intensity and duration, nevertheless, some common symptoms appear as overall reduction in vegetative biomass, reproductive growth and yield-traits. The effects of heat and drought stresses may differ, when applied singly; for example, heat stress during seed filling may accelerate or even suppress the seed filling process, and reduce the duration of filling to inhibit the accumulation of various reserves (Prasad et al., 2008b; Chakrabarti et al., 2013). Drought stress slowed down the seed filling process due to water limitations, but eventually resulted in inhibitory effects on seed size and numbers (Pushpavalli et al., 2014; Sehgal et al., 2017). While these two stresses affect the seed filling differently, each one leads to reduced seed size due to reduction in the cell number in endosperm/cotyledons, inhibitions or acceleration of seed filling rate and various biochemical processes, as in wheat (Nicolas et al., 1985) and maize (Monjardino et al., 2005). Variations may also exist in the relative composition of seed reserves in response to either of these stresses, nevertheless, in general, seed quality was adversely affected by both the stresses (Khan et al., 2004; Spiertz et al., 2006; Thuzar et al., 2010; Krishnan et al., 2011; Rashid et al., 2017).

The combined effects of heat and drought stress are an excellent example of two different abiotic stresses occurring in the field simultaneously (Shah and Paulsen, 2003; Barnabás et al., 2008). Little is known about on the combined effects of heat and drought stress on crops, with most studies reporting very severe effects on crop growth and productivity (Cairns et al., 2013; Hamidou et al., 2013; Awasthi et al., 2014; Sehgal et al., 2017). The impact of the simultaneous occurrence of two stresses is more pronounced during early part of reproductive processes, especially micro-and mega-sporogenesis, pollen and stigmatic function, anthesis, pollination, pollen tube growth, fertilization, and early embryo development, compared to individual drought or heat stress (Prasad et al., 2008b). Failure in any of these processes will drastically decrease the fertilization rate or leads to early embryo abortion, thus resulting in lower seed or grain numbers and finally limiting the crop yield (Prasad et al., 2008b). Substantial losses observed in crop yields under combined stress environment have been attributed to several reasons, such as metabolic changes, reductions in the period of crop developmental stages (Reddy et al., 2004). Subsequent decline in light perception, decreased phenology, and perturbation of processes involved in carbon assimilation such as transpiration, photosynthesis and respiration may contribute to fewer, malformed and smaller seeds (Barnabás et al., 2008; Awasthi et al., 2014, 2017), which are economically futile.

Seed-filling duration declines more in combined stresses than individual treatments, as evidenced in wheat (Shah and Paulsen, 2003; Farooq et al., 2017a,b), chickpea (Awasthi et al., 2014) and lentil (Sehgal et al., 2017). Both the stresses can occur simultaneously after anthesis to limit seed-filling duration, and result in poor-quality grains in wheat (Wardlaw, 2002; Farooq et al., 2017a), due to the substantial reduction in seed dry weight, seed numbers, and starch content (Balla et al., 2011). The rate of transport of non-structural carbohydrates in endosperm tissue decreases, as in wheat, in response to dual heat and drought stress (Wardlaw, 2002; Plaut et al., 2004). There is severe reduction in starch accumulation due to combined stresses imposed during seed filling, which has been attributed to more drastic inhibition of starch synthesizing enzymes, compared to individual stress treatments, as in chickpea (Awasthi et al., 2014) and lentil (Sehgal et al., 2017), consequently seeds become shriveled. Combined heat and drought stress can reduce nitrogen pool due to a decline in free amino acids containing various transfer substances related to metabolism of nitrogen and other osmotic compounds (Prasad et al., 2008b; Awasthi et al., 2014). The response to dual stress situation is crop specific, for example, in soybean plants, subjected to dual stress, seeds showed higher protein contents, but lower oil contents than controls/individual stresses, (Dornbos and Mullen, 1992). In cereals such as barley and wheat, the combined stress treatment reduced starch accumulation but increased protein content, as compared to single stress treatment (Savin and Nicolas, 1996). In *Brassica* species, exposed to combined heat and drought stress, seed protein content increased while seed weight decreased at higher temperature (35/18°C), in comparison to moderate temperature (28/18°C) (Gan et al., 2004). Considering that heat and drought stress are frequently experienced together and would be a major risk for the grain crops in future, more studies are needed to evaluate the impacts of these two stresses on mechanisms at various organizational levels affecting seed yield and quality in various crops.

## Integrative ‘Omic’ Approaches and Molecular Mapping on Seed Filling in Response to Drought and Heat Stress

Though, seed filling stage is one of the most important phases that determine yield, based on the available information regarding the stress sensitivity of enzymatic processes involved in accumulation of starch, the complete picture is missing. The individual and combined effects of heat and drought stress on crop production and yield is a complex phenomenon that includes processes as diverse as assimilation and supply of nutrients to various reproductive organs, accumulation of stem reserves, gamete formation, fertilization and embryo development, and endosperm and seed development. Developmental studies, if integrated with “omic” studies would determine the complex gene expression pattern during seed filling and unravel the molecular basis of the impacts of heat and drought stress on seed quality and composition (Figures 2, 3). To deduce the extremely susceptible molecular process underlying the seed filling phenomenon and the effect of drought and heat stress episode(s) on seed filling, different approaches involving genomics, transcriptomics, proteomics, micromics and epigenomics are imperative to identify not only the key responsible genes but also other regulatory molecules/proteins influencing make them responsive to genetic improvement.

## Transcriptomics and Seed Filling

Using microarray technology in rice, ∼21,000 genes, which are involved in the successive stages of grain filling, have been identified, and a majority of them relate to the metabolic pathways of carbohydrate and fatty acids (Zhu et al., 2003). Cluster analysis and correlation studies reveal the association of 269 genes with grain filling (Zhu et al., 2003). The information about the rice genome sequence has helped in the recognition of promoter regions that control these genes, and has led to the discovery of common *cis*-elements shared in the promoter region in the grain filling genes. Using grain gene cluster-analysis, it has been revealed that AACA element appears to be dominant among 103 available promoters. These eventually led to deciphering nine transcription factors that help in regulating gene expression (Zhu et al., 2003). Study in the Barley caryopses using Affymetrix 22K Barley1 Gene Chip (Close et al., 2004), at 21 days post-anthesis stage (Mangelsen et al., 2011) reveal that 2020 genes were differentially expressed under heat stress. The genes with a role in biosynthesis of storage compounds and cell growth are down-regulated indicating disruption of seed development. Further, genes for production of sugars increased, which provide evidence for high production of compatible solutes as well as feed-back induced substrate accumulation for biosynthesis of storage compounds (Mangelsen et al., 2011). Metadata analysis in the same study reveals that embryo and endosperm are the primary targets of heat stress response (Mangelsen et al., 2011). The impact of high temperature on grain filling during the milky stage of rice has been elucidated by Yamakawa et al. (2007). The genes involved in starch synthesis, GBSS and SBE show decreased expression while the starch consuming α-amylase show increased expression. In rice caryopses, exposed to high temperature, the transcription level of genes encoding for an enzyme ADP-Glc pyrophosphorylase (AGPase) declines, but not in the SSS isoforms levels. The variations noticed in transcript levels were correlated to the observed biochemical differences between the starch grains formed both under normal and high temperatures, mainly, with reduced amylose content, side chain elongation of amylopectin and smaller grain size.

Similar observations have been reported when drought stress is applied during the first few days after pollination, where inhibition of endosperm division occurs which is related to reduced kernel size at maturity, such as in maize (Ober et al., 1991) and wheat (Nicolas et al., 1985). The endosperm and placental/pedicel tissues of maize, drought-stressed for 5–9 days after pollination, have been examined using cDNA microarray (Yu and Setter, 2003). A marked difference occurs in the response of both tissue types: in the pedicel, 89% of the 79 transcripts affected show up-regulation whereas, in the endosperm, 82% of the 56 transcriptionally altered genes show down-regulation. In case of pedicel, transcription levels of stress-related genes [e.g., heat shock proteins (HSPs) and chaperone genes] are enhanced whereas, in the endosperm, the genes involved in cell division, cell wall degradation and growth are down-regulated. In another study, the expression of starch and sucrose metabolism pathway genes like *a-Amy3* gene encoding α-amylase, and an alpha-glucosidase (*ONG2*), catalyzing the hydrolysis of the raw starch granules, in wheat is altered during drought stress at grain filling stage (Ma et al., 2017).

## Micromics of Seed Filling

Beside the transcriptomics, micromics approach also provides information about the post-transcriptional gene regulation of the seed filling through identification of different microRNAs (miRNAs). The role of miRNAs as master regulators in controlling the gene expression has been studied in various crop species (Jin et al., 2015). Different studies have identified the crucial role of miRNAs and their regulation in seed filling process through their target identification in different crop species like rice, wheat and maize, etc. (Peng et al., 2013; Yi et al., 2013; Jin et al., 2015). Members of miR156 family show exclusive expression during grain filling process in rice by targeting the squamosal promoter binding protein like (*SPL*) family genes. *SPL16* regulates the cell proliferation at the time of grain filling in rice and the increased expression of *SPL16* is directly proportional to the grain yield (Wang et al., 2012). The role of SPLs in different yield related traits like grain size, grain quality, yield, panicle branching, tillering and plant height has been described in rice (Si et al., 2016). Overexpression of miR397 shows its role in grain size by down-regulating its target, *lac* that encodes laccase protein (Zhang et al., 2013). Likewise, increased expression of miR156, miR164, miR166, miR167, and miR1861 during grain development and grain filling stages signifies their regulatory roles (Peng et al., 2013). Studies about the influence of miRNAs during grain filling under heat and drought stress periods are very limited. In one such study in wheat, the expression of miRNAs- miR159a, 159b, miR160, and miR171a during heat stress at grain filling stage decreases (Goswami et al., 2014). More studies are needed to understand the finer role of micro RNAs involved in regulation of seed filling under drought as well as heat stress.

## Proteomics-Based Studies on Seed Filling

Proteomic reference maps are available for various crops during grain filling and maturation stages, e.g., wheat (Vensel et al., 2005) and maize (Méchin et al., 2004) endosperm and barley grain (Finnie et al., 2002). Exploring the individual as well as combined impacts of drought and heat stress on protein composition can be useful for improving protein quality in crops. Heat stress effects have been studied thoroughly on hexaploid wheat grains at the protein levels (Majoul et al., 2003, 2004) where various proteins involved in starch metabolism decrease whereas HSPs increase (Majoul et al., 2004). Subsequently, differentially 121 proteins are reported to reveal considerable alterations in response to drought stress in the proteome of wheat grain, (Hajheidari et al., 2007), of which 57 have been identified. More than half of the proteins identified are thioredoxin targets, unveiling the link between drought and oxidative stress. Another study on wheat, exposed to heat stress during grain filling stage (Wang et al., 2015) show increase in expression of proteins involved in signal transduction, photosynthesis, antioxidant enzyme, ATP synthase, HSPs, and other nitrogen metabolism related proteins in tolerant genotype, as compared to sensitive genotypes, indicating their crucial role in tolerance (Wang et al., 2015). Proteomics studies in the rice grain development show the association and acquisition of different metabolic pathway proteins including glycolysis, citric acid cycle, lipids and proteins in the mature grains (Mitsui et al., 2013). Assessment of seeds developing under drought or/and heat in contrasting genotypes would reveal vital information about the type of transcripts and proteins influenced by stresses, which would be beneficial for their genetic manipulation to improve stress tolerance.

## QTLs Associated With Seed Filling

‘Stay green’ trait can be a convenient trait to use as an indicator of sustainable supply of assimilates and utilization of stem reserves, which can also be regarded as a mutually exclusive strategy to promote seed filling under stressful conditions (Blum, 1998; Abdelrahman et al., 2017). Many QTLs (Xu et al., 2000; Abdelkhalik et al., 2005; Harris et al., 2007) or candidate genes (Lee et al., 2001; He et al., 2005; Gregersen and Holm, 2007) related to leaf senescence have been identified in cereals and might prove crucial for genetic modifications in breeding. Five QTLs in two bread wheat genotypes are identified on chromosomes 1B, 2B, 3B, 5A, and 6B under high-temperature stress during grain filling (Sharma D. et al., 2016). A QTL for the heat susceptibility index for the grain filling period is present in close association with QTL region for productive tillers under late-sown conditions and grain filling duration on chromosomes 1B and 5A, respectively. Similarly, a major QTL, qDTY1.1 is present on chromosome 1 for grain yield for terminal drought stress in rice. This shows the positive effect on grain yield during drought stress conditions (Vikram et al., 2011). Another major QTL on linkage group 2 (LG 2) for terminal drought tolerance, identified in pearl millet, is also considered as a marker for grain yield improvement across different terminal stress conditions. Thus, these QTLs can be used for molecular wheat breeding programs for conferring heat as well as drought tolerance (Sharma D. et al., 2016).

## Conclusion

Seed-filling processes are adversely affected by heat and drought stress in all crop species, resulting in poor-quality seeds and reduced seed yields. The frequency of these two stresses occurring at the same time is increasing, for both for summer- and cool-season crops, which is highly detrimental to the qualitative and quantitative aspects of yield. Hence, future research should focus on investigating the dual effects of drought and heat, involving various physiological, biochemical and molecular approaches. Future endeavors should also pay attention on screening the existing germplasm of various crops under combined stress environment to identify tolerant genotypes and their subsequent incorporation into breeding programs. It is vital to understand and dissect various components influencing seed-filling processes under separate and combined stress environments to unveil varying responses of different crops to these two stresses. Identification of sensitive sites (embryonic stages, hormonal changes and biochemical pathways for seed reserves, signaling molecules, proteins and genes) related to seed-filling processes in stressed plants, especially under the combined stress, would provide useful cues in developing strategies to improve seed quality. As photosynthetic activity mainly determines crop productivity, the breeding for ‘stay-green’ trait is essential to combat drought as well as heat stress. ‘Omics’ studies are in progress that will be useful for identifying the genes, proteins, and metabolites in developing seeds that are, impacted by heat or drought stress. Modeling the stages of development, growth, grain productivity, grain quality and sink-source relations will enable better insights on the physiological and genetic nature of stress tolerance, ultimately resulting in enhanced grain yields and quality in crops. Improved models can enhance the likelihood of predicting crop performance in future challenging climates, which will largely help to identify traits that can be exploited through breeding to produce sustainable climate-resilient genotypes with acceptable yield under stressed environments.

## Author Contributions

All authors listed have made a substantial, direct and intellectual contribution to the work, and approved it for publication.

## Conflict of Interest Statement

The authors declare that the research was conducted in the absence of any commercial or financial relationships that could be construed as a potential conflict of interest.
